# Genome Wide Identification and Characterization of Apple bHLH Transcription Factors and Expression Analysis in Response to Drought and Salt Stress

**DOI:** 10.3389/fpls.2017.00480

**Published:** 2017-04-11

**Authors:** Ke Mao, Qinglong Dong, Chao Li, Changhai Liu, Fengwang Ma

**Affiliations:** State Key Laboratory of Crop Stress Biology for Arid Areas, College of Horticulture, Northwest A&F UniversityYangling, China

**Keywords:** apple, genome-wide analysis, bHLH transcription factor, expression analysis, drought and salt stress, regulation networks

## Abstract

The bHLH (basic helix-loop-helix) transcription factor family is the second largest in plants. It occurs in all three eukaryotic kingdoms, and plays important roles in regulating growth and development. However, family members have not previously been studied in apple. Here, we identified 188 MdbHLH proteins in apple “Golden Delicious” (*Malus* × *domestica* Borkh.), which could be classified into 18 groups. We also investigated the gene structures and 12 conserved motifs in these *MdbHLH*s. Coupled with expression analysis and protein interaction network prediction, we identified several genes that might be responsible for abiotic stress responses. This study provides insight and rich resources for subsequent investigations of such proteins in apple.

## Introduction

Because they are sessile, plants are inevitably affected by environmental factors from which they cannot escape. To optimize their growth and survival, they must utilize a wide range of physiological and biochemical processes in their responses to diverse abiotic stresses (Feller et al., 2011; Sun et al., 2015). Those responses are coordinated by the regulation of gene expression (Agarwal et al., 2006; Pires and Dolan, 2010; Feller et al., 2011). Transcription factors (TFs) play crucial roles in these processes by activating or repressing related downstream genes (Agarwal et al., 2006; Feller et al., 2011; Rehman and Mahmood, 2015). These TF families (Pérez-Rodríguez et al., 2010) include MYBs (Jin and Martin, 1999), bHLHs (basic helix-loop-helix; Massari and Murre, 2000), DREBs (dehydration responsive element-binding; Agarwal et al., 2006), ERFs (ethylene responsive element binding factor; Rehman and Mahmood, 2015), bZIPs (basic region/leucine zipper motif; Corrêa et al., 2008), and WRKY members (Zhang and Wang, 2005), which represent the six major gene families that control stress responses (Kavas et al., 2016).

The bHLH superfamily is the second largest TF family in plants (Feller et al., 2011). Since the discovery of the bHLH motif during research on murine muscle development (Murre et al., 1989), this superfamily has been found in all three eukaryotic kingdoms (Carretero-Paulet et al., 2010). The family is defined by the bHLH domain, which comprises approximately 60 amino acids and contains two functionally distinct regions: the basic region and the HLH region (Toledo-Ortiz et al., 2003). The basic region, with 13–17 amino acids, is located at the N-terminus of the bHLH domain, where it binds the specific E-box (CANNTG) DNA sequence. In addition, this region has six typical basic residues and a highly conserved HER motif (His 5-Glu 9-Arg 13) that are predicted to bind specific DNA sequences (Feller et al., 2011; Kavas et al., 2016). In contrast, the HLH region is composed of two amphipathic α-helices linked by a loop of variable length (Murre et al., 1989; Ferré-D'Amaré et al., 1994). This region functions as a dimerization domain (Massari and Murre, 2000).

As the sequencing of genomes for more species is completed, numerous plant bHLH proteins are being identified and characterized (Pires and Dolan, 2010; Feller et al., 2011; Song et al., 2014; Xu et al., 2014; Chen et al., 2015; Sun et al., 2015; Zhang et al., 2015; Kavas et al., 2016). However, such studies are relatively limited when compared with research efforts with animal species (Song et al., 2014; Kavas et al., 2016). Based on evolutionary relationships, DNA-binding specificity, and conservation of specific amino acids and domains (in addition to the bHLH domain), the animal bHLH proteins have been organized into six groups, A through F (Atchley and Fitch, 1997). Then these bHLH proteins are further divided into small subfamilies (Ledent and Vervoort, 2001; Simionato et al., 2007). In plants, however, the number of groups has not been determined but is thought to cover 15–26 groups (Buck and Atchley, 2003; Pires and Dolan, 2010). Phylogenetic analyses of some atypical bHLH proteins have even extended that number to 32 (Carretero-Paulet et al., 2010). In addition to this bHLH domain, various additional motifs and amino acids are conserved in different bHLH subgroups (Carretero-Paulet et al., 2010; Pires and Dolan, 2010; Feller et al., 2011).

Most of the bHLH proteins already identified and functionally characterized in plants are AtbHLH proteins (Carretero-Paulet et al., 2010), and only a few bHLH family genes have been examined in apple (*Malus* × *domestica*; Xie et al., 2012; Qu et al., 2016). Results from studies of plant bHLH-coding genes have suggested that they are involved in regulating a diverse array of biological and biochemical processes, such as light signaling (Roig-Villanova et al., 2007; Leivar et al., 2008), hormone signaling (Friedrichsen et al., 2002; Lee et al., 2006), shoot branching (Komatsu et al., 2001), stomata, and root development (Ohashi-Ito and Bergmann, 2007; Kanaoka et al., 2008), and abiotic stress responses (Chinnusamy et al., 2003; Kiribuchi et al., 2004).

Under stress conditions, some special bHLH TFs are activated and bind to the promoter of the key genes involved in various signaling pathways, and regulate the stress tolerance of plants by regulating the transcription level of these target genes. For example, in response to low temperature, AtICE1 binds to the MYC recognition site (CANNTG; a kind of E-box) in the promoter sequence of *CBF3* gene, and induces the expression of *CBF3*, which functions to increase the resistance of *Arabidopsis* to cold stress (Chinnusamy et al., 2003). AtNIG1, which was recognized as the first calcium binding TF involved in salt stress signaling pathway, specifically binds to the E-box element in promoter region of some salt stress related genes, and modulates the tolerance to salt stress by regulating downstream gene expression (Kim and Kim, 2006). AtMYC2 promotes the transcription of the drought responsive gene *RD22* by binding to its promoter, and thereby increases the drought resistance of *Arabidopsis* (Abe et al., 2003). Besides, a recent study indicates a direct interaction between AtMYC2 and the ABA receptor AtPYL6, which is inhanced by the presence of ABA, and makes MYC2 important in ABA-JA crosstalk (Aleman et al., 2016). In apple, studies of bHLH TFs are mainly focused on fruit coloration in recent years (An et al., 2012, 2016; Xie et al., 2012; Hu et al., 2016), and just few findings have been reported on the regulation of stress tolerance of *MdbHLH* genes. Feng et al. (2012) identified an AtICE1 like protein from apple, named MdCIbHLH1. It could bind to the MYC recognition site in the promoter region of a DREB family gene *MdCBF2*, and regulates apple plants resistance to low temperature by up-regulating the expression of *MdCBF2*. In addition, MdbHLH104 was proved to enhance the tolerance to iron deficiency by promoting the transcription of *MdAHA8* (Zhao et al., 2016).

Even though so many important functions have been recognized, and sequencing of the apple genome has been completed (Velasco et al., 2010) and now improved (Li et al., 2016), no detailed analysis has previously been reported for bHLH family proteins in apple. In this study, we identified and characterized 188 bHLH family genes in apple. They are located on 17 different chromosomes and can be classified into 18 main groups based on phylogenetic analysis. We also investigated their gene structures and conserved domains and motifs. Expression of *Arabidopsis* homologous genes and *MdbHLH*s was also analyzed in plants under drought and salt stress, and we predicted the possible interacting proteins and regulatory networks for genes related to those stress responses. Our goal was to provide a useful resource for subsequent research on the functions and regulatory mechanisms of potentially important MdbHLH proteins that are crucial in modulating abiotic stress responses in apple.

## Materials and methods

### Sequence retrieval and identification of bHLH proteins in apple

We downloaded the file for genome-wide protein sequences in apple (Malus_x_domestica.v1.0.conensus_peptide.fa.gz) from the Genome Database for Rosaceae (GDR; https://www.rosaceae.org/). The Hidden Markov Model (HMM) file of the HLH domain (PF00010) was downloaded from the Pfam database (version 30.0; http://pfam.xfam.org/; Finn et al., 2016), and was used as a query to scan the proteome file via HMMER software (version 3.1b2; http://hmmer.org/) with a default *E*-value. The protein sequences for genes shown in those HMMER results were obtained from the proteome file using a Bioperl script, and were submitted to the SMART database (http://smart.embl-heidelberg.de) and the online Batch CD-search tool (https://www.ncbi.nlm.nih.gov/Structure/bwrpsb/bwrpsb.cgi; Marchler-Bauer et al., 2015) to verify the existence of the conserved bHLH domain. Redundant sequences were removed with online ElimDupes software (http://hcv.lanl.gov/content/sequence/ELIMDUPES/elimdupes.html), and a few sequences with obvious errors were removed manually.

### Chromosomal locations, characterizations, and functional annotation of bHLH genes in apple

The GFF file (Malus_x_domestica.v1.0.consensus.gff.gz), containing location data and annotation information for apple genes, was downloaded from the GDR database. MapInspect software was used to map the apple bHLH genes on different chromosomes, and annotation data in the GFF file were exhibited by the online tool WEGO (http://wego.genomics.org.cn/cgi-bin/wego/index.pl). The lengths, masses, isoelectric point (pI)-values, and charge at pH7.0 for these bHLH protein sequences were determined with DNAstar software, and length distributions and functional annotations were analyzed with Blast2GO software (version 2.8.0).

### Phylogenetic analysis of apple bHLH proteins

The protein sequences of putative bHLH genes in apple were aligned with ClustalX software (version 1.83) with default parameters, and phylogenetic trees were constructed using the aligned result with MEGA6 software (version 6.06; Tamura et al., 2013) via the Neighbor-Joining (NJ) method (Parameter setting: Bootstrap method-1,000 replicates, Poisson model, Pairwise deletion). Two versions of that tree were produced: the original tree and a bootstrap consensus tree.

### Analysis of gene structure (intron-exon) and identification of conserved motifs

Information about the gene structure (intron-exon) of each putative bHLH gene was obtained from the GFF file, downloaded from the GDR database. The schematic structures of these genes were drawn with the online Gene Structure Display Server (GSDS 2.0; http://gsds.cbi.pku.edu.cn). Local MEME software (4.11.2; Bailey and Elkan, 1994) was used to identify conserved motifs in the protein sequences according to the following parameters: -protein, -oc m12, -mod zoops, -nmotifs 12, -minw 6, and -maxw 70. The results from these analyses of gene structure and conserved motifs were arranged according to the order shown on the phylogenetic tree.

### Expression analysis of homologous genes in *Arabidopsis* under drought and salt stress conditions

The *Arabidopsis* proteome file (TAIR10_pep_20101214) was downloaded from the TAIR database (http://www.arabidopsis.org). Using each putative MdbHLH protein sequence as a query, we performed a local blast (blastp method) to find the homologous genes for these bHLH proteins in *Arabidopsis*, using BioEdit software (version 7.0.9.0). We also obtained transcriptome data (Affymetrix microarray data; Kilian et al., 2007) for *Arabidopsis* treated with drought (GSE5624) or salt (GSE5623) stress from the National Center for Biotechnology information (NCBI) database (http://www.ncbi.nlm.nih.gov/). After ID conversion (GenBank ID and Affymetrix ID) and data extraction, the expression patterns of these orthologs under different conditions were displayed in the form of heatmaps (log_2_-transformed) that were drawn with HemI software (version 1.0.1; Deng et al., 2014). Cluster analysis was conducted with OmicShare tools, a free online platform (www.omicshare.com/tools). The average value of gene expression at each time point was transformed with log_2_ method, and parameter settings are as follows: number of clusters 5; *p* < 0.05; fold change > 2.

### Protein association network predictions and functional annotations by string

All of the putative MdbHLH protein sequences were submitted to the online server STRING (version 10.0; http://string-db.org), with the organism specified as “*Arabidopsis thaliana*.” After the blast step was finished, genes with the highest scores (Bitscore) were used to construct the network. Genes that did not interact with any others were removed. The functional annotation information was copied manually from the blast results.

### Plant materials and growth conditions

Tissue-cultured apple seedlings (Gala) were grown on MS medium with 0.5 mM 6-BA and 0.1 mM IAA, and grown in controlled environment cabinets under 16 h light/8 h dark conditions at 22°C unless stated otherwise.

Experiments involving drought or salt stress were conducted in a controlled environment chamber. Tissue-cultured apple seedlings were transferred to rooting medium (MS + 0.1 mM IAA) for rooting. Thirty days later, select the plantlets with consistent growth state and transferred into hydroponic conditions (Hoagland nutrient solution) for 7 days. Then these plantlets were treated for various time periods with PEG6000 (10%; W/V) to simulate drought conditions or NaCl (200 mM) to induce salt stress, under continuous white light conditions (Osram L18W/30 tubes; 20 μmol m^−2^s^−1^). Roots of these plantlets were sampled at specified time points, frozen in liquid nitrogen and saved in −80°C for subsequent use.

### RNA extraction and real-time quantitative RT-PCR analysis

Plant materials were harvested, frozen in liquid nitrogen, and then ground under RNase-free conditions. The RNA was extracted with TRizol reagent (Invitrogen), following the manufacturer's instructions, and then treated with DNase I at 37°C for 30 min. The RNA (1 μg) was then reverse-transcribed using a PrimeScript First-strand cDNA Synthesis Kit (Takara) according to the manufacturer's instructions. A 10-μL aliquot of cDNA was diluted to 100 μL with water, and 2 μL of that diluted cDNA was used for the analyses.

For real-time quantitative RT-PCR (qRT-PCR), gene-specific primers for our selected *MdbHLH*s were designed and synthesized by Sangon Biotech (Shanghai) Company (product size 110–130 bp; Tm 59–61°C; details are shown in Supplementary Table S1), and *Md18S* was used as an internal control. All reactions were performed on an Icycler iQ5 system (Bio-Rad), using the SYBR Green Supermix Kit (Bio-Rad) according to the manufacturer's instruction. Expression levels of these genes were calculated as 2^−ΔCT^ values (expression level and standard deviation data were shown in Supplementary Table S2), and heatmaps (Supplementary Figure S6) were drawn with HemI software (log_2_-transformed). Besides, relative expression levels for each gene along the time series were also calculated as 2^−ΔΔCT^ values (**Figure 5**). At least three biological replicates were used for the fluorescence-quantitative PCR reactions, with each biological repeat having at least three technical replicates. Each biological repeat contains at least 6 plantlets for mixing. Spearman correlation analysis between the expression patterns of *MdbHLHs* in apple and *MdbHLH* orthologs in *Arabidopsis* were performed using R softwares.

## Results

### Identification, chromosomal locations, and functional annotation of apple bHLH genes

**Figure 1:**
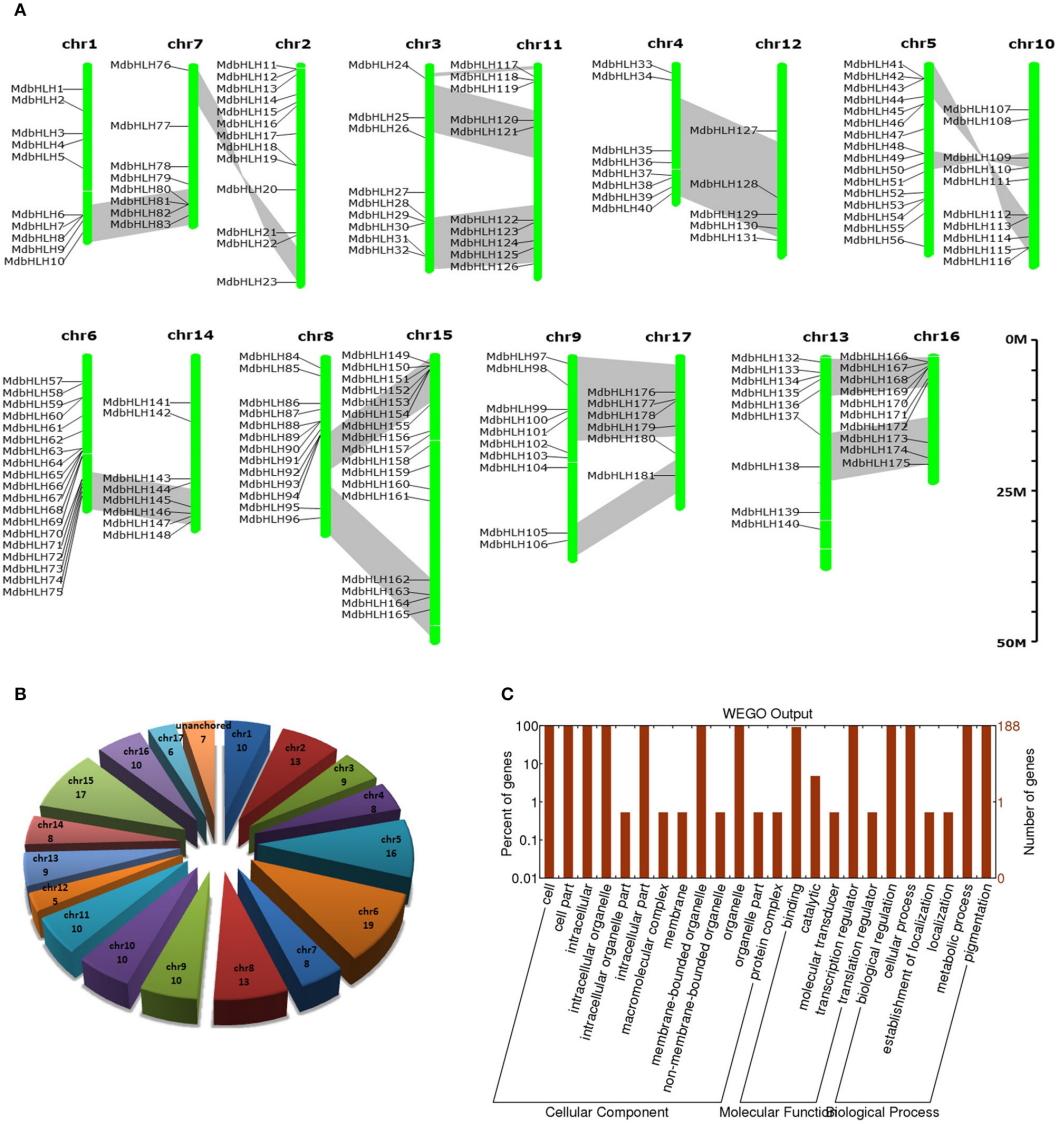
**Summary information for the 188 MdbHLH genes. (A)** Locations of 188 *MdbHLH*s on 17 chromosomes. **(B)** Distribution of 188 *MdbHLH*s on chromosomes. **(C)** Functional annotation (Gene Ontology) of MdbHLH proteins. Segmented duplicate homologous blocks are indicated with gray shadow.

For genome-wide identification of MdbHLH genes, we used the HMM file as a query to search the apple proteome. With default parameters, 253 putative MdbHLH protein sequences were obtained. After the existence of the conserved bHLH domain was confirmed by SMART and CD-Search, and redundant sequences were removed, we were able to identify 188 sequences as genes in the apple bHLH family. These were named *MdbHLH1* to *MdbHLH188*, based on their chromosomal locations (Figure 1A; Supplementary Table S3). Sequence analysis revealed that these MdbHLH proteins vary widely in length, from 128 to 963aa, and have predicted molecular weights of 14,666–105,632 Da. Their predicted pI-values range from 4.51 to 10.10. Gene IDs, genomic positions, charge at pH 7.0 values, and information about Gene Ontology (GO) annotations were also summarized for these MdbHLH proteins (Supplementary Table S3).

Based on the genomic position information obtained from the GDR database, we showed that the *MdbHLH* genes were found across all the 17 apple chromosomes, ranging from 5 to 19 per chromosome. However, the other seven *bHLHs* (MdbHLH182-MdbHLH188) were localized to unassembled genomic scaffolds, and can not be mapped to any particular chromosome according to what we currently know about this genome (Figures 1A,B; Supplementary Table S3). Chromosome 6 has the most *MdbHLH*s (19 total), followed by chr 15 (17 genes) and chr 5 (16 genes). Furthermore, although chr 12 is longer than either chr 5 or chr 6, it contains only five *MdbHLH*s, making it the least populated with genes in that family (Figures 1A,B).

The GDR database provided GO annotation information about these MdbHLH proteins (Supplementary Table S3), which we were able to depict by using WEGO software (Figure 1C). There are three aspects of function classifications: CC (cellular component), MF (molecular function), and BP (biological process). The MF and BP aspects mainly described the molecular activities of multiple gene products, and included associated pathways and processes that are enriched by these genes, i.e., binding, transcription regulation, biological regulation, cellular process, metabolic process, and pigmentation. All of these functions and processes are closely related to TFs. In addition, the enrichment results for CC aspects (where gene products are active) were consistent with the primary roles that TFs have. Because the annotation information obtained from the GDR database was simple, we sought more details by running an annotation analysis with Blast2GO software. Those results indicated that individual MdbHLH proteins are 200–600 aa long (average 404 aa; Supplementary Figure S1). These GO annotation results were largely similar to what we obtained from the GDR database. For example, in the MF aspect, the MdbHLH proteins were mainly enriched in binding activities, especially protein-binding and sequence-specific DNA-binding (Supplementary Figure S1; Supplementary Table S4), both of which are the main mechanisms by which bHLH family TFs regulate the expression of downstream genes (Leivar et al., 2008; Feller et al., 2011; Xie et al., 2012).

### Phylogenetic analysis, display of gene structure, and prediction of conserved motifs

**Figure 2:**
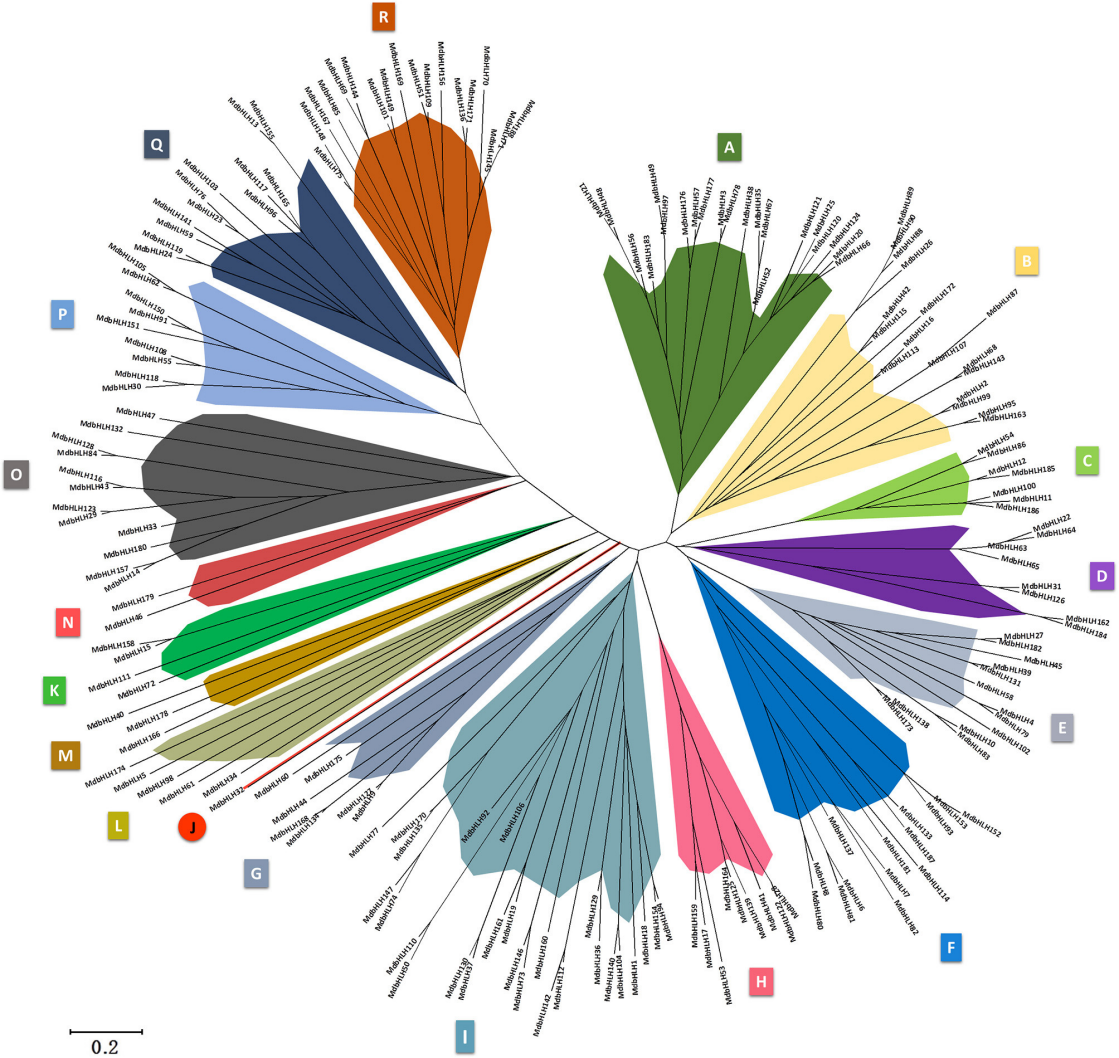
**Phylogenetic analysis (original tree) and subgroup classifications of MdbHLH proteins**.

The exact number of subgroup classifications for plant bHLH proteins is unknown, but is thought to be 15–32 (Buck and Atchley, 2003; Carretero-Paulet et al., 2010; Pires and Dolan, 2010). To evaluate the evolutionary relationships of our 188 proteins, we conducted a phylogenetic analysis based on full-length protein sequences. Applying the NJ method, we assigned the proteins to18 main groups: A through R (Figure 2; Supplementary Figure S2), and then subdivided each of the three largest groups (A, I, and R) into two subgroups (Supplementary Figure S2). This resulted in 21 MdbHLH subgroups, which is the same number as those found in tomato (Sun et al., 2015) and *Phaseolus vulgaris* (Kavas et al., 2016). Unlike the other groups in this phylogenetic tree, Subgroup J contained a single bHLH protein, meaning that *MdbHLH32* is also unique (Figure 2). The five smallest groups were J through N, each having one to five MdbHLHs (Figure 2; Supplementary Figure S2).

The evolutionary relationships among these MdbHLH proteins were also determined by analyzing their gene structures and conserved motifs. These composition patterns tended to be consistent with the results from our phylogenetic tree, being nearly identical among genes within the same group, but varying greatly between groups. Except for Motifs 1 and 2, which were conserved in all MdbHLH proteins (Supplementary Figures S3, S4), some of the other 10 motifs were present only in certain groups, which might explain why functions for MdbHLH proteins tend to be specific to a particular group. These included Motifs 4 and 8, only present in Groups A and D; Motifs 7 and 9, in Group A; Motif 6, Groups D and E; Motif 11, Group E; Motif 10, Group R; Motif 3, mainly present in Groups P, Q, and R, except for MdbHLH40, which is in Group M; and Motif 12, mainly present in Groups O and Q but with two exceptions: MdbHLH5 in Group L and MdbHLH160 in Group I (Supplementary Figure S3). Overall, Group A had the most motifs, and the genes in other groups had more complex gene structures.

Although most MdbHLHs within a group showed similar composition patterns, obvious differences were noted for three genes: *MdbHLH151* in Group P, *MdbHLH13* in Group Q, and *MdbHLH70* in Group R. Their genomic sequences were much longer than those of others within the same group. For example, *MdbHLH151* had eight introns while most of the others had only four. That gene also lacked the first specific exon that was contained in all of the remaining genes in Group P, and which was also conserved in some other groups (Supplementary Figure S3). For *MdbHLH13*, its long genomic sequence and protein sequence were very different from others in Group Q. The MdbHLH13 protein also had an additional motif (Motif 12) that was mainly conserved in Group O, and was present only in MdbHLH13 and MdbHLH155 in that group (Supplementary Figure S3). The MdbHLH70 protein lacked Motif 2, which was conserved in 181 of the 188 MdbHLH proteins (Supplementary Figures S3, S4). In addition to these three, MdbHLH176 lacked both Motifs 1 and 2, making it an atypical bHLH protein. Meanwhile, even though MdbHLH152 did have those two motifs, their locations were reversed, with Motif 2 being in the front (Supplementary Figure S3).

### Expression profiling and regulatory network predictions for *MdbHLH* orthologs in *Arabidopsis*

**Figure 3:**
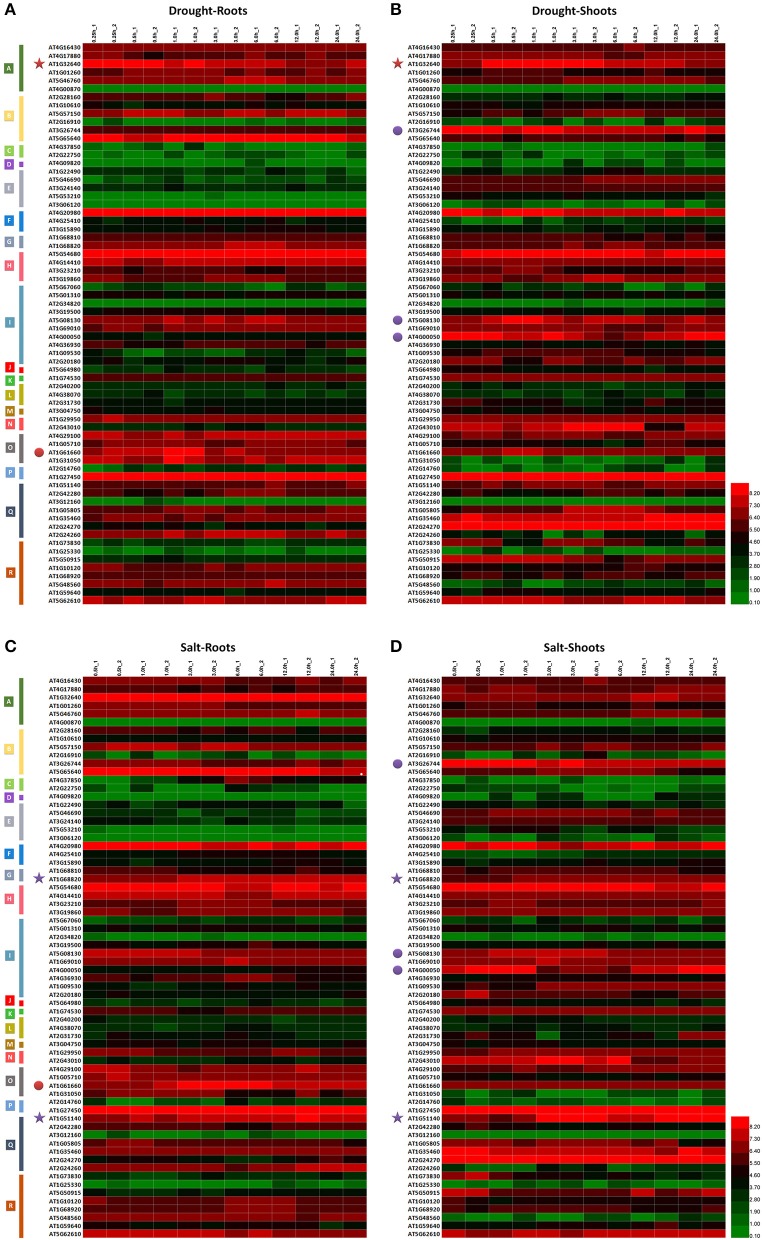
**Expression analysis of ***MdbHLH*** orthologs in ***Arabidopsis*****. Expression patterns of *MdbHLH* orthologs under drought **(A,B)** and salt **(C,D)** stress. Different colors indicate genes with expression patterns that are similar between tissue types (stars) or treatments (dots).

For our preliminary investigation of the functions of MdbHLH genes in regulating plant responses to abiotic stress, we downloaded the transcriptome data of homologous genes in *Arabidopsis* (Supplementary Table S5), and evaluated their expression patterns (Figure 3; Supplementary Figure S5). Overall, expression of many *AtbHLH*s was significantly different among tissue types, regardless of which stress conditions were tested (Figure 3). This suggested that their expression was tissue-specific. In contrast, some genes showed different (or even opposite) expression patterns (Figure 3; Supplementary Figure S5), indicating that their responses varied according to the stress conditions applied.

Several TF genes demonstrate coordinated regulation of responses to drought and salt stress (Jin and Martin, 1999; Agarwal et al., 2006; Feller et al., 2011; Kavas et al., 2016). We also observed this here in responses to drought or salt, as well as in having similar expression patterns in different tissues and/or under different treatment conditions (Supplementary Figure S5). This was all evidence for how essential those genes are in regulating plant responses. For example, under drought conditions, AT1G32640 was down-regulated in both roots and shoots whereas AT1G51140 and AT1G68820 were up-regulated significantly under salt stress (Figure 3). Expression of both AT1G61660 and AT5G08130 under drought or salt stress increased initially in the roots and shoots, respectively, before being down-regulated, whereas AT4G00050 was first down-regulated before expression increased under those same test conditions (Figure 3; Supplementary Figure S5; Supplementary Table S5). These results also suggested that some genes in bHLH family may utilize opposing regulatory mechanisms under abiotic stresses.

**Figure 4:**
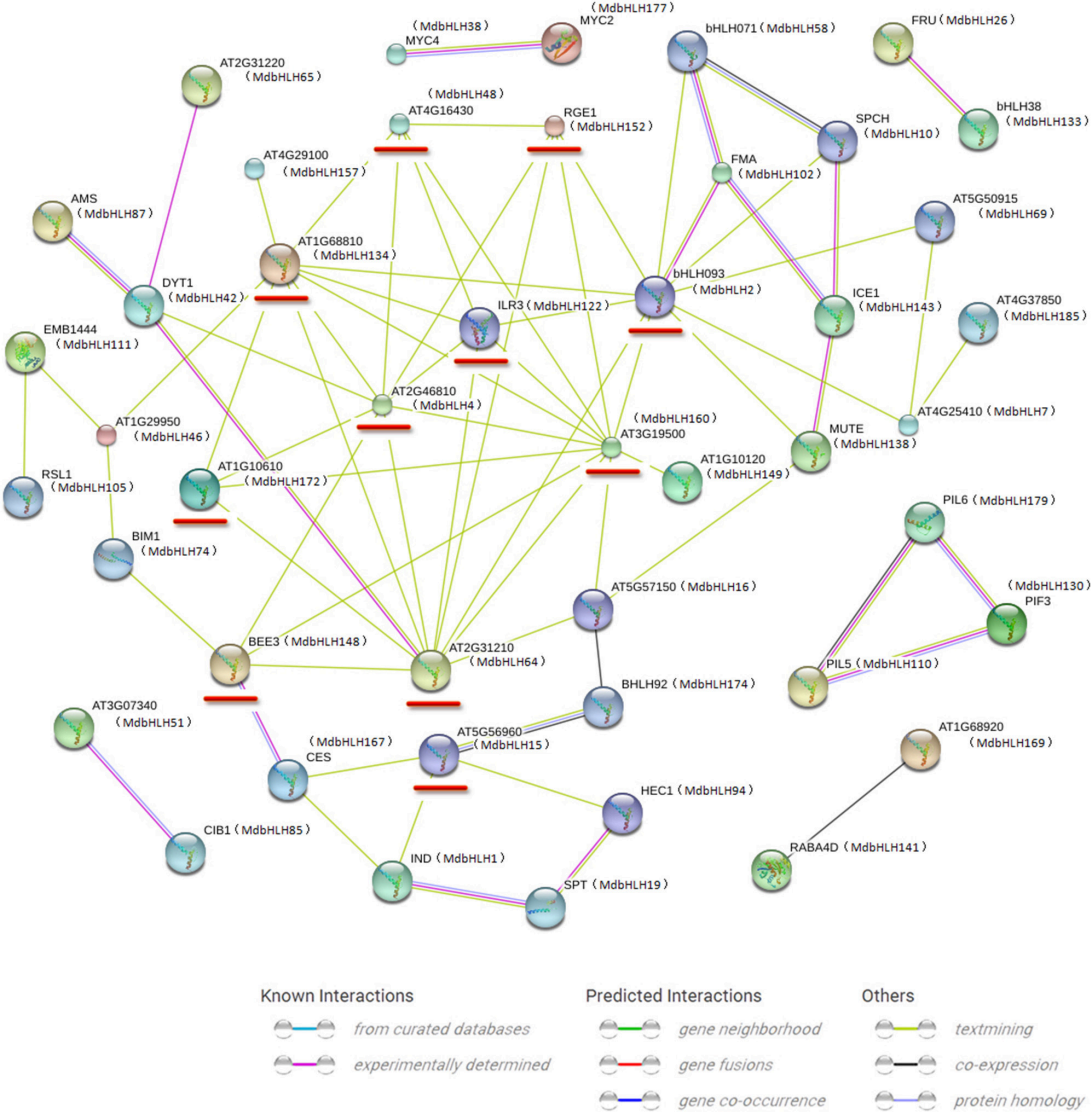
**Protein interaction network for MdbHLHs based on MdbHLH orthologs in ***Arabidopsis*****. This network was predicted by online software STRING. Red lines indicate proteins that are predicted to interact with more than four other bHLH proteins. MdbHLH proteins are shown in brackets with *Arabidopsis* orthologs.

Our STRING approach allowed us to predict a protein interaction network using the 188 MdbHLH protein sequences as queries. This produced a complex regulatory network constructed with orthologs in *Arabidopsis*. Many bHLH proteins interacted with more than one bHLH (Figure 4), which is in accord with previous reports that binding activity with specific DNA sequences depends upon the formation of homodimers or heterodimers of different bHLH proteins (Pires and Dolan, 2010; Feller et al., 2011). Overall, we found 11 proteins that could interact with more than four other bHLH proteins, making them important players in regulating plant growth and stress responses (Supplementary Table S6). Furthermore, our blast results strongly supported our previous classifications of the MdbHLH proteins (Supplementary Table S6). Detailed information about these orthologs is also summarized in Supplementary Table S6.

### Expression analysis of *MdbHLH* responses to salt and drought stress

To identify which of these bHLH genes are most important in the response to abiotic stresses, we studied changes in expression for 30 *MdbHLH*s (Supplementary Table S7). These were selected because they demonstrated unique composition patterns, and because of their roles in stress responses or in protein interaction networks, as deduced from the expression analysis and predicted regulatory network for homologous genes in *Arabidopsis*. Besides, correlation analysis (spearman) between the expression patterns of selected *MdbHLH* genes in apple and *MdbHLH* orthologs in *Arabidopsis* were also performed. Because of lacking the expression data for four hmomolgue genes (*MdbHLH4*-AT2G46810, *MdbHLH15*-AT5G56960, *MdbHLH64*-AT2G31220, and *MdbHLH152*-AT1G49770) in the downloaded file (Supplementary Table S8), we performed a correlation analysis of the remaining 26 genes.

**Figure 5:**
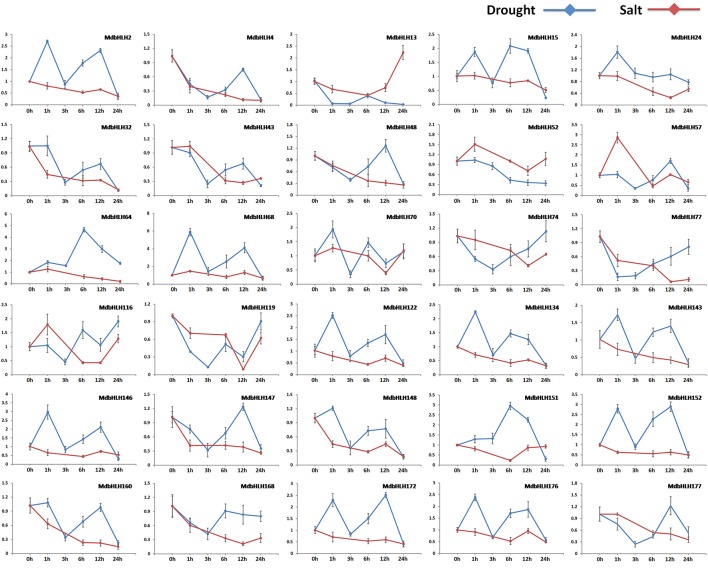
**Relative expression analysis of select genes along the time series**. Analysis (2^−ΔΔCT^ method) of 30 selected *MdbHLH*s expressed in response to induced drought (PEG6000; blue line) or salt stress (NaCl; red line).

Expression for most of these genes was significantly altered in the early stage of drought or salt treatment (Figure 5; Supplementary Figure S6). Among them, some showed changes in expression that were similar or opposite between drought and salt treatments. For example, under both of those stresses, *MdbHLH74* and *MdbHLH119* were initially down-regulated significantly before being up-regulated. Their responses tended to be more rapid under drought conditions, usually changing within the first hour (Figure 5; Supplementary Figure S6). The opposite was noted for *MdbHLH24* and *MdbHLH151*. Under drought stress, both were up-regulated at first and then down-regulated, whereas, under salt stress, their expression initially decreased before increasing (Figure 5; Supplementary Figure S6). Some other genes showed changes in expression that were specific to the type of treatment applied. They included *MdbHLH4, MdbHLH168*, and *MdbHLH177*, which were down-regulated under salt stress. Under drought conditions, however, their expression was also down-regulated at first, but then increased (Figure 5; Supplementary Figure S6). Other genes were significantly down-regulated by salt treatment but responded in different ways to drought, including *MdbHLH2 MdbHLH15, MdbHLH32, MdbHLH43, MdbHLH48, MdbHLH64, MdbHLH77, MdbHLH134, MdbHLH143, MdbHLH147, MdbHLH148, MdbHLH152, MdbHLH160*, and *MdbHLH172* (Figure 5; Supplementary Figure S6). Among them, *MdbHLH143* is exactly the cold-induced bHLH TF gene named *MdCIbHLH1* (Feng et al., 2012; Supplementary Table S3), which we have mentioned in introduction. In addition to expression analysis, correlation analysis between *MdbHLH* genes and their orthologs in *Arabidopsis* found that several showed a high correlation, such as *MdbHLH24, MdbHLH52, MdbHLH74, MdbHLH119, MdbHLH134*, and *MdbHLH143*, with *p* < 0.05 (Supplementary Table S8; Supplementary Figure S7). Moreover, the expression patterns of *MdbHLH134* and its ortholog (*AtbHLH30*, AT1G68810) showed a significant correlation (*p* < 0.01), no matter under drought or salt conditions (Supplementary Table S8).

### Development of protein interaction networks for *MdbHLH*s that are crucial to the abiotic stress response

Proteins in the bHLH family function primarily by forming homodimers or heterodimers with other proteins, actions that are vital for their binding to related downstream genes (Toledo-Ortiz et al., 2003; Carretero-Paulet et al., 2010; Pires and Dolan, 2010). As part of our preliminary study of the regulatory mechanism(s) of important MdbHLH genes under abiotic stress, we predicted the protein interaction networks for *MdbHLH24* and *MdbHLH74*, which demonstrated either similar or opposite expression patterns under different treatment conditions (Figure 5). We also examined *MdbHLH4*, which showed a significant response to drought and salt treatments (Figure 5). Besides, its homologous gene, AT2G46810, was located in the center of the predicted gene association network (Figure 4).

**Figure 6:**
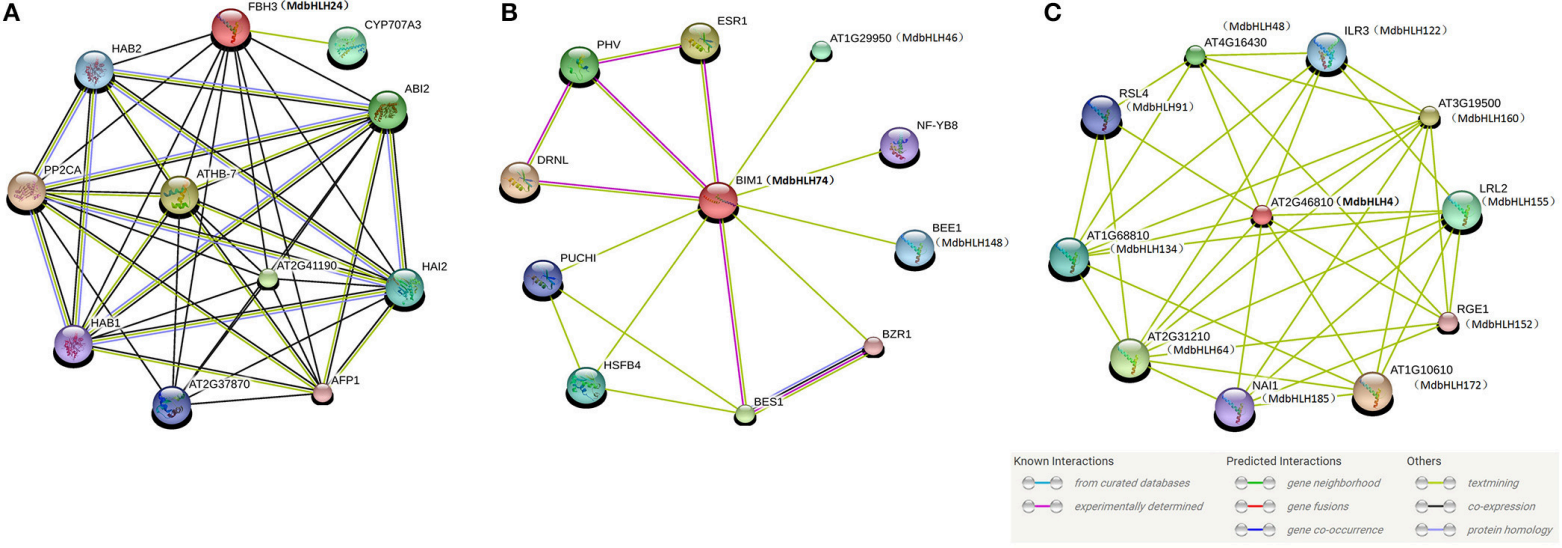
**Protein interaction networks prediction for crucial MdbHLH proteins**. Protein interaction network predictions of MdbHLH24 **(A)**, MdbHLH74 **(B)**, and MdbHLH4 **(C)**, based on MdbHLH orthologs in *Arabidopsis*. MdbHLH proteins are shown in brackets with *Arabidopsis* orthologs.

Although the predicted network for MdbHLH24 (FBH3 ortholog) was mainly obtained based on text-mining and co-expression analysis (Figure 6A), most of the other proteins were involved in ABA signaling pathways, such as ABI2, HAB1, and 2, PP2CA, and CYP707A3 (Supplementary Table S9). The ABA signal has an important role in regulating the plant response to various abiotic stresses, such as drought, salt, and cold (Agarwal et al., 2006; Rehman and Mahmood, 2015). Therefore, this result was consistent with our previous deduction that *MdbHLH24* is a crucial regulator of stress responses in apple, and that these interacting proteins provide useful resources for subsequent research. Moreover, our investigation of MdbHLH74 (ortholog of BIM1) showed that it plays central roles in regulating various proteins with different functions, several of which have been experimentally determined (Figure 6B; Supplementary Table S9). This suggested that, in addition to controlling the abiotic stress response, MdbHLH74 regulates many other aspects of plant growth and development, such as modulating shade avoidance in apple when coupled with BEE1 (Cifuentes-Esquivel et al., 2013). Our findings were again in accord with the various functions identified already for bHLH family genes, especially when combined with genes in other families (Corrêa et al., 2008; Feller et al., 2011; Rehman and Mahmood, 2015). Finally, the results of two predicted networks (Figures 4, 6C) indicated that MdbHLH4 (ortholog of AT2G46810) has crucial roles in the bHLH family and that most of the proteins that possibly interact with MdbHLH4 are also members of that family (Figure 6C; Supplementary Table S9). This implied that MdbHLH4 interacts with more bHLH proteins than do other bHLHs, such as MdbHLH24 and MdbHLH74. Although our network suggested that MdbHLH4 has a wider range of regulatory functions, most genes in this network (Figure 6C) have not been functionally characterized in *Arabidopsis*, including At2G46810 (*AtbHLH70*), the *MdbHLH4* ortholog (Supplementary Table S9). However, we now recognize that future examinations can begin with *MdbHLH4* as part of an extended focus on the potential functions and mechanisms of bHLH family genes in apple.

## Discussion

Completion of apple genome sequencing (Velasco et al., 2010; Li et al., 2016) has enabled resarchers to identify and characterize many TF genes at the whole-genome level, including MYB (Cao et al., 2013), ERF (Girardi et al., 2013), DREB (Zhao et al., 2012), bZIP (Wang et al., 2015), and WRKY (Meng et al., 2016). However, no such detailed studies have been done with the bHLH family, and only a few examinations have been made of *MdbHLH*s (An et al., 2012, 2016; Xie et al., 2012). Here, we identified 188 MdbHLH genes in apple. Expression analysis and predictions of interaction networks allowed us to identify genes that have crucial roles in the apple response to salt and drought stresses.

### Classification of MdbHLH proteins based on polygenetic relationship, gene structure, and conserved motifs

Based on phylogenetic analysis, intron-exon organizations, DNA-binding motifs, predicted conserved protein motifs, and amino acids, we classified our 188 MdbHLH proteins into 18 groups and then further divided Groups A, I, and R into two subgroups each, such that the total number was 21. This is the same number reported for tomato (Sun et al., 2015), even though that organism has fewer SlbHLHs (159) than the 188 MdbHLHs in apple. Our phylogenetic tree (Figure 2) indicated that Group A has two main branches. The MdbHLH genes in subgroup A-2 have two prominent and similar exons, making them different from A-1. Besides, the patterns of motif composition showed that the MdbHLH proteins in A-2 do not contain Motif 9. Therefore, MdbHLH38 more likely belongs to the A-1 subgroup, which is different from A-2 on the phylogenetic tree (Supplementary Figure S3). If one considers that a significant distance exists between MdbHLH38 and other A-2 proteins (Figure 2), and that this protein has a gene structure and motifs similar to those of other A-1 proteins, then we might propose that MdbHLH38 represents a transitional form in the evolution of those two subgroups. Likewise, if one takes into account the more complex but consistent motif composition patterns of the A-2 subgroup, we can conclude that it might evolved from the A-1 subgroup, thereby acquiring some specific regulatory functions that are common to many large TF families (Zhang and Wang, 2005; Agarwal et al., 2006; Feller et al., 2011; Rehman and Mahmood, 2015). Based on these findings and suppositions, we suggest that *MdbHLH38* is a useful gene for studying evolutionary processes within Group A, and it may even provide a model for investigations of other groups within the bHLH family. With regard to Groups I and R, their very obvious differences in gene structure between Subgroups I-1 and I-2, as well as the fact that R-2 contains the specific Motif 10 that R-1 is lacking, means that those particular subgroup classifications are much easier to make.

### Identification of important MdbHLH genes that may regulate responses to drought and salt stress in apple

To determine which of the 188 *MdbHLH*s are the most important regulators of stress responses in apple, we studied how the expression of their orthologs in *Arabidopsis* was changed when plants were exposed to drought or saline conditions (Figure 3; Supplementary Figure S5). Regulating the functions of bHLH proteins depends upon the formation of homodimers or heterodimers between bHLH proteins, or between bHLH and non-bHLH proteins (Herold et al., 2002; Hernandez et al., 2007). Thus, for genes that show similar expression patterns (Supplementary Figure S5), we might conclude that they likely interact with each other at the protein level (Leivar et al., 2008), and also coordinately control the expression of downstream genes (Feller et al., 2011). Those regulatory mechanisms also depend upon the co-expression of these interacting genes. Nevertheless, it is also possible that the expression of one bHLH gene can be affected, or even regulated, by another one, such that the two appear to have similar changes in expression. Further research is needed to explore the interrelationships between such genes.

We also found here that some *MdbHLH* genes showed contrasting changes in expression in response to the same stress treatment. Feller et al. (2011) have reported that a single bHLH protein can interact with more than one bHLH protein and some are even able to interact with several bHLHs or non-bHLH proteins. For example, the bHLH protein PIF3 can homodimerize or heterodimerize with PIF4, and also can heterodimerize with an atypical bHLH factor HFR1 (Castillon et al., 2007). And the MYB-bHLH-WDR (MBW) complex (Feller et al., 2011) may be the best proof for the type of interaction between the bHLH family and other families. In *Arabidopsis*, studies of the bHLH TF gene *AtTT8* showed that there is a positive feedback mechanism in regulation of self expression, which is depends on the MBW complex (Baudry et al., 2006). The *in vivo* binding of TT2 and PAP1 to the *TT8* promoter is dependent on the simultaneous expression of *TT8*, which is crucial for further induction of *TT8* expression (Baudry et al., 2006). For PIFs, PIF4, and PIF5 can form homodimers and bind to the promoter of target genes in dark conditions (Xu et al., 2015). Under light conditions, however, another bHLH TF factor HFR1 will directly inhibits these PIFs by forming non-DNA-binding heterodimers with PIF4 and PIF5 (Hornitschek et al., 2009). If we consider the opposite expression patterns that we detected here between different *bHLH* genes, as well as their complex mechanisms for protein interactions, then we might conclude that the feedback mechanism should be responsible. In other words, some bHLH genes may specifically respond to stress conditions and form heterodimers with specific bHLH proteins, leading to the differentiation in the expression between different bHLH genes. Further research is needed to verify this hypothesis.

Because various bHLH proteins respond differently to environmental factors, we can hypothesize that genes located in the center of regulatory networks are more important, such as *MdbHLH4* (AT2G46810) and *MdbHLH134* (AT1G68810; Figure 4). In *Arabidopsis*, the bHLH TF ABS5/T5L1/bHLH30 (ortholog of MdbHLH134; Supplementary Table S6) is essential for leaf morphogenesis, and related to auxin signaling (An et al., 2014). Besides, expression analysis of *AtbHLH30* (260034_at) in roots showed that, although did not respond to drought, it was significantly down-regulated by salt stress treatment (Figure 3; Supplementary Table S8). These diverse functions are consistent with its central location in the regulatory network (Figure 4). In this study, the expression pattern of *MdbHLH134* and *AtbHLH30* showed a significant correlation in spearman correlation analysis (*p* < 0.01) under drought and salt conditions (Supplementary Table S8; Supplementary Figure S7). Based on this extremely similar expression pattern, and the hypothesis that orthologous proteins are likely to perform similar or identical functions in other organisms (Feller et al., 2011), we can infer that *MdbHLH134* should play important roles in leaf development and salt stress response in apple, and may be realted to auxin.

Although *MdbHLH143* (*MdCIbHLH1*) is not located in the center of regulatory networks, its expression is down-regulated by salt treatment obviously (Supplementary Figure S6), and its expression pattern exhibited a significant negative correlation with its ortholog *AtICE1* under drought conditions (Supplementary Figure S7; Supplementary Table S8). In *Arabidopsis, AtICE1* encodes a MYC-like bHLH TF protein that binds to Myc recognition sequences in the *CBF3* promoter under cold stress conditions (Chinnusamy et al., 2003). Following studies in other organisms suggest that, except for cold, ICE1-like genes may also play impotant roles in regulating other plant stress resistance (Chen et al., 2012; Feng et al., 2013), such as drought and salt, by regulating the expression of *DREB1* (*CBF*) genes (Wang et al., 2008; Yang et al., 2011). These results suggest that *MdbHLH143* (*MdCIbHLH1*) should not only regulates plant response to cold (Feng et al., 2012), but also to drought and salt stresses. Besides, the *AtMYC2* orthologous gene *MdbHLH177* exhibited a significant response to drought and salt conditions (Supplementary Figure S6), indicating a impotrant role of this gene in regulating stress resistance in apple, just like *AtMYC2* in *Arabidopsis* (Abe et al., 2003; Aleman et al., 2016).

### Regulatory network predictions of crucial MdbHLH genes that show significant responses to drought and salt stress

Based on our analyses, we selected some *MdbHLH*s for further expression analysis under drought and salt stress. As we had expected, most of them exhibited significant responses and showed different patterns of expression depending on the type of treatment applied, such as *MdbHLH4, MdbHLH24*, and *MdbHLH74* (Figure 5). Actually, except lacking the expression data of MdbHLH4 orthologous gene, expression of both *MdbHLH24* and *MdbHLH74* showed significant correlation with their orthologous genes, respectively (Supplementary Figure S7; Supplementary Table S8). These results suggest that these genes play important roles in apple stress response. We also used *Arabidopsis* orthologs to predict the regulatory networks for these three representative genes, *MdbHLH24, MdbHLH74*, and *MdbHLH4*. For the first two, their functions and regulatory mechanisms matched those of their orthologs (Supplementary Table S9), and conincide with the assumption that they are crucial for regulating apple tolerance to drought and salt stresses. For example, most proteins that are predicated to interact with MdbHLH24 are involved in ABA signaling pathway (Supplementary Table S9), which is crucial for abiotic stress response in plants (Agarwal et al., 2006). This result also suggests that MdbHLH24 regulates plant stress tolerance by regulating the expression of key genes in the ABA signaling pathway.

However, most of the proteins that interact with MdbHLH4 have not yet been characterized, even in *Arabidopsis*. Nonetheless, because the expression of *MdbHLH4* is induced by drought and salt treatments significantly, and its ortholog is located in the center of the network and can interact with many bHLH proteins that belong to different signaling pathways, we believe that this protein has a wide range of regulatory roles in apple, and may be a critical component of competitive regulatory mechanisms. The functional annotation of the three known proteins (RGE1, NAI1, and ILR3) also supports this hypothesis (Supplementary Table S9). These three genes are involved in regulating different aspects of plant growth and development, suggesting the extensive functions for proteins that interact with MdbHLH4. The functions and mechanisms of MdbHLH4 and its interacting proteins are largely unknown, but provide a good opportunity for researchers to reveal the potential operations of such mechanisms for bHLH proteins in general, and the unique functions of apple MdbHLH proteins in particular.

In conclusion, this report is the first to describe the identification of 188 bHLH family genes in apple. In addition to investigating their functions and structures, we also performed expression analyses and developed a regulatory network to determine which genes are most active for stress responses in this crop species. Improving our understanding of the competitive regulatory mechanisms of bHLH proteins, and the genes that are central to this proposed mechanism, means that we now have rich resources for subsequent studies of gene cloning and functional characterization of members in this *MdbHLH* family.

## Author contributions

The study was conceived by FM. KM and QD collected the public datasets for Apple and the other researched species. KM, QD, CLi, and CLiu contributed to data analysis, bioinformatics analysis and manuscript preparation. KM, QD, and CLi participated in the qRT-PCR experiment. FM, KM, and CLiu participated in the planning of experiments and revising the manuscript.

### Conflict of interest statement

The authors declare that the research was conducted in the absence of any commercial or financial relationships that could be construed as a potential conflict of interest.

## Supplementary material

Supplementary Figure S1 **Annotations of 188 MdbHLH proteins performed by Blast2GO. (A)** Length distribution of sequences. **(B–D)** Annotations for CC, MF, and BP components, respectively.

Supplementary Figure S2 **Phylogenetic analysis (circle tree) and subgroup classifications (21 subgroups) of MdbHLH proteins**.

Supplementary Figure S3 **Analysis of gene structure and conserved motifs for MdbHLH proteins**. Phylogenetic tree **(A)**, organization of gene structure **(B)**, and distribution of conserved motifs **(C)** for 188 MdbHLH proteins.

Supplementary Figure S4 **Motif logos for 12 conserved motifs**. The motif logos were predicted by local MEME software. Red stars indicate conserved HER motif (His 5- Glu 9-Arg 13).

Supplementary Figure S5 **Cluster analysis of gene expression data for ***MdbHLH*** orthologs (fold change >2) in ***Arabidopsis*** under drought or salt treatment**.

Supplementary Figure S6 **Expression analysis of selected ***MdbHLH*** genes**. Analysis (2^−ΔCT^ method) of 30 *MdbHLH*s expressed in response to induced drought (PEG6000) **(A)** or salt stress (NaCl) **(B)**.

Supplementary Figure S7 **Correlation analysis between the expression patterns of selected ***MdbHLH*** genes and ***MdbHLH*** orthologs in ***Arabidopsis***, under drought (A)** or salt **(B)** conditions. The value of spearman correlation coefficent in the heatmap was multiplied by one hundred. Positive or negative correlation relationships between the gene expression patterns are indicated with positive or negative values. Color depth (blue or red) of the grids corresponds to the value.

Supplementary Table S1 **qRT-PCR primers used in this study**.

Supplementary Table S2 **The expression level and standard deviation data for selected MdbHLH family genes**.

Supplementary Table S3 **Chromosomal location and functional annotation of 188 apple bHLH genes**.

Supplementary Table S4 **GO annotation for MdbHLH proteins**.

Supplementary Table S5 **Expression level for ***MdbHLH*** orthologs in ***Arabidopsis*** under different stress conditions**.

Supplementary Table S6 **Summary information for 188 MdbHLH proteins in STRING database**. The predicted important bHLH TFs involved in regulating plant growth and stress responses were marked in red and listed in this table (sheet3).

Supplementary Table S7 **Name list of the selected 30 ***MdbHLH*** genes for subsequent expression analysis**.

Supplementary Table S8 **Correlation analysis between the expression patterns of selected ***MdbHLH*** genes and ***MdbHLH*** orthologs in ***Arabidopsis*****. Genes with significant correlation are marked with asterisks (* for *p* < 0.05; ** for *p* < 0.01).

Supplementary Table S9 **Summary information for proteins that may interact with MdbHLH24, MdbHLH74, or MdbHLH4**.
