# Supplementary figures and images for: Genome Wide Identification and Characterization of Apple bHLH Transcription Factors and Expression Analysis in Response to Drought and Salt Stress
Source: Front Plant Sci. 2017 Apr 11;8:480. doi: 10.3389/fpls.2017.00480 (PMC5387082; doi:10.3389/fpls.2017.00480)

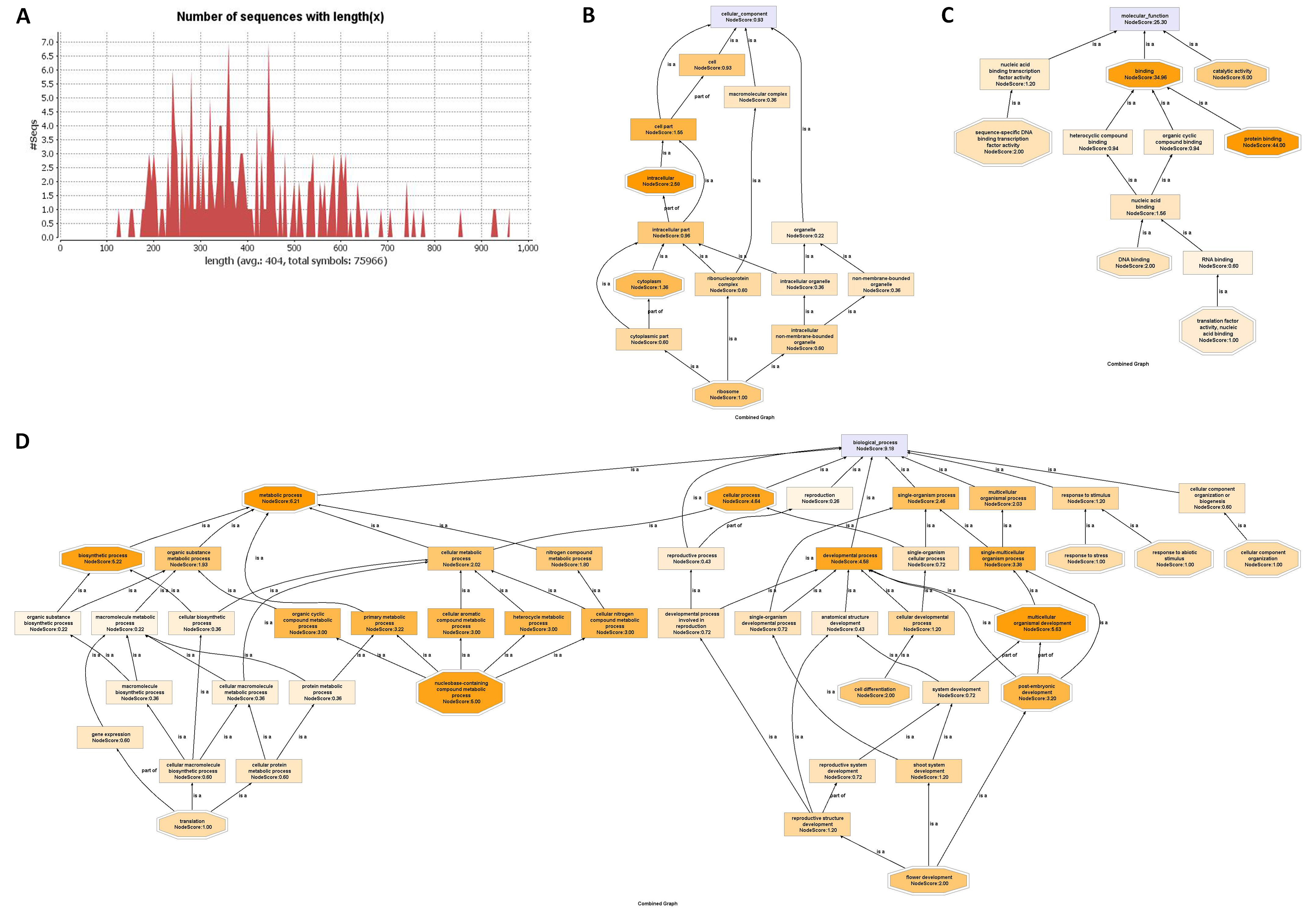

Supplement: Supplementary Figure S1 — Annotations of 188 MdbHLH proteins performed by Blast2GO. (A) Length distribution of sequences. (B–D) Annotations for CC, MF, and BP components, respectively. [file Image1.JPEG]

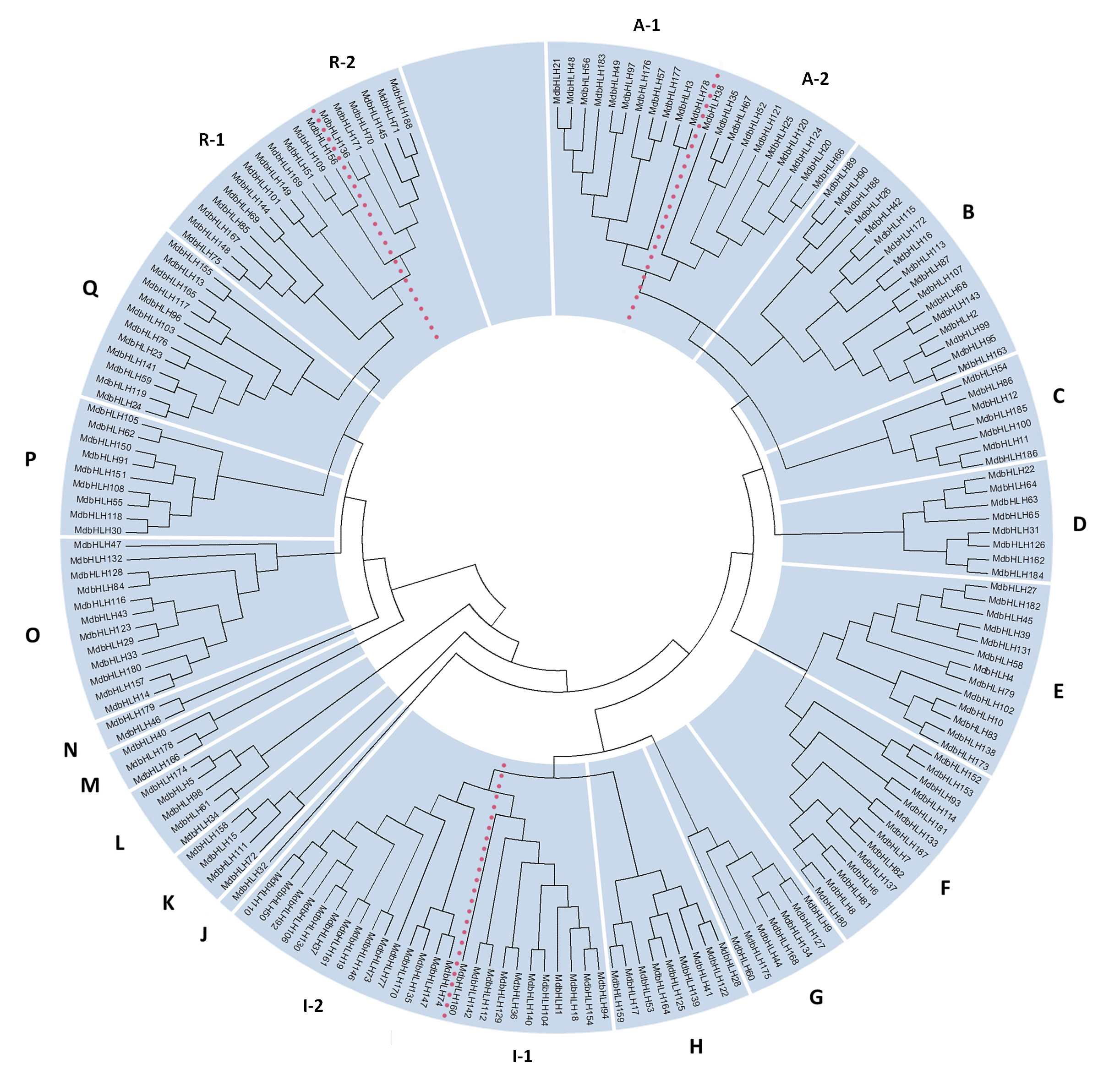

Supplement: Supplementary Figure S2 — Phylogenetic analysis (circle tree) and subgroup classifications (21 subgroups) of MdbHLH proteins. [file Image2.JPEG]

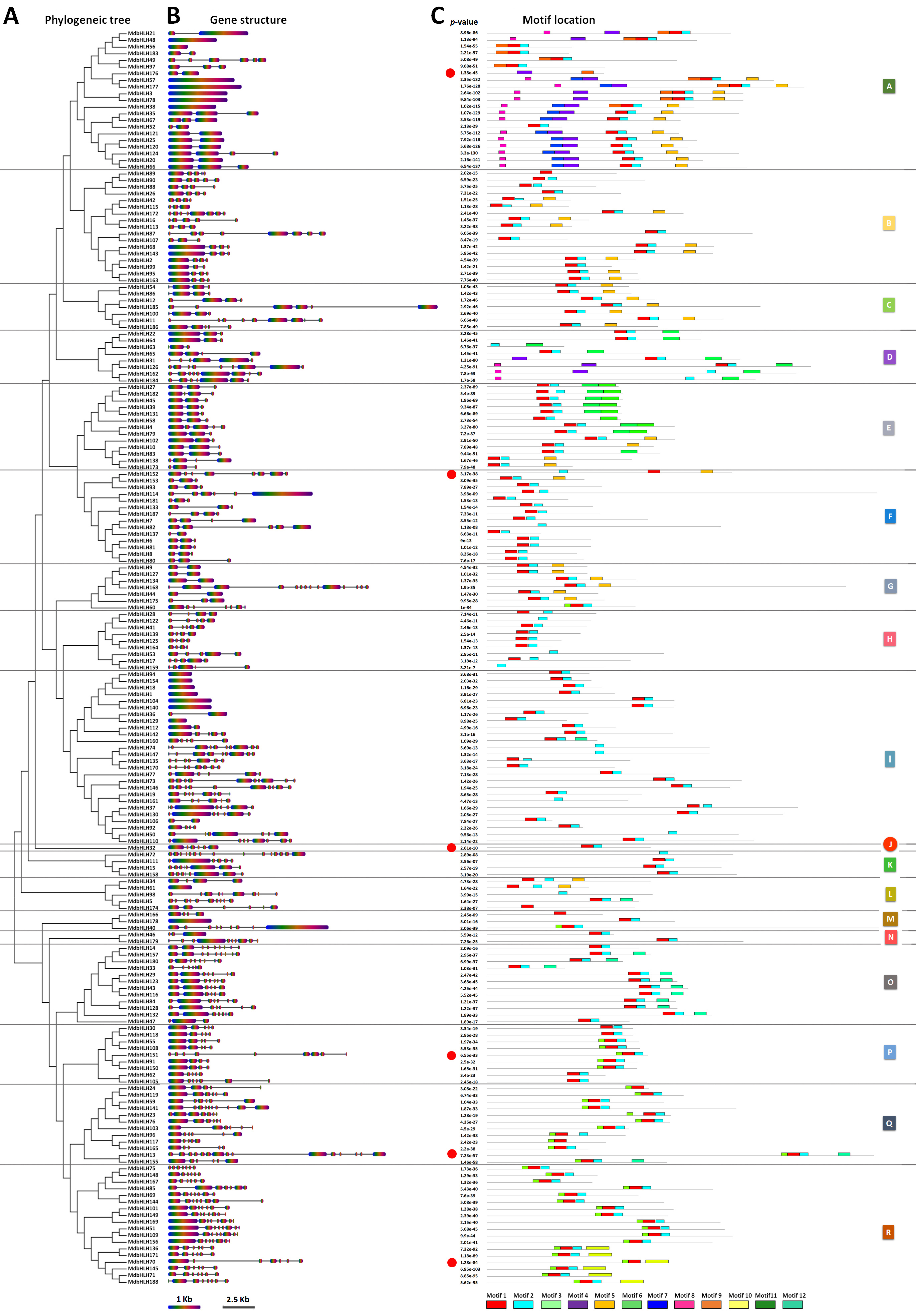

Supplement: Supplementary Figure S3 — Analysis of gene structure and conserved motifs for MdbHLH proteins. Phylogenetic tree (A), organization of gene structure (B), and distribution of conserved motifs (C) for 188 MdbHLH proteins. [file Image3.JPEG]

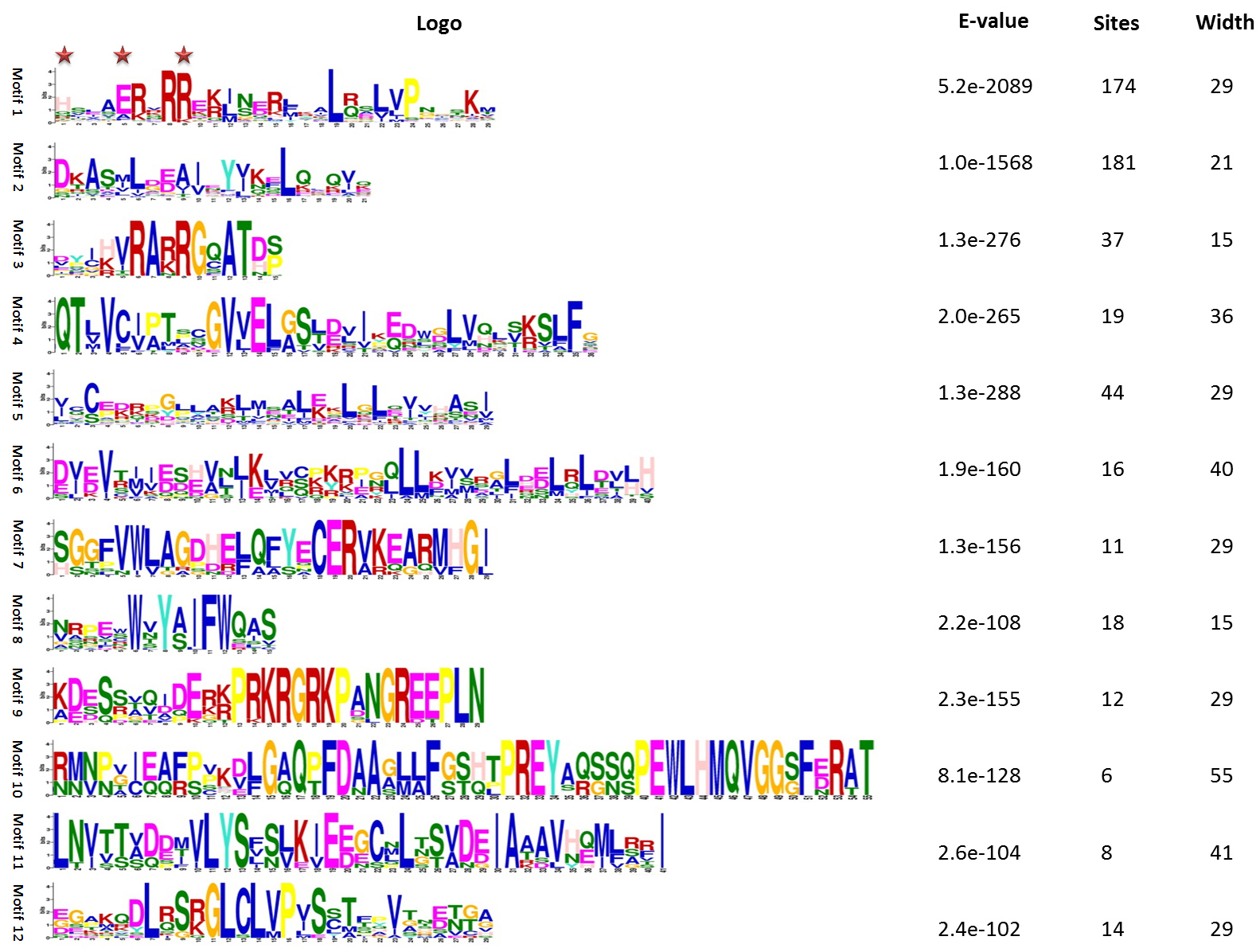

Supplement: Supplementary Figure S4 — Motif logos for 12 conserved motifs. The motif logos were predicted by local MEME software. Red stars indicate conserved HER motif (His 5- Glu 9-Arg 13). [file Image4.JPEG]

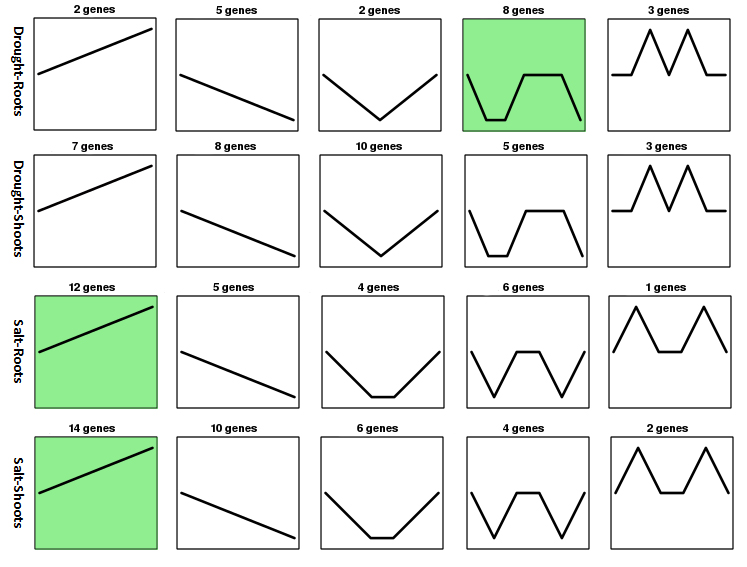

Supplement: Supplementary Figure S5 — Cluster analysis of gene expression data for MdbHLH orthologs (fold change >2) in Arabidopsis under drought or salt treatment. [file Image5.JPEG]

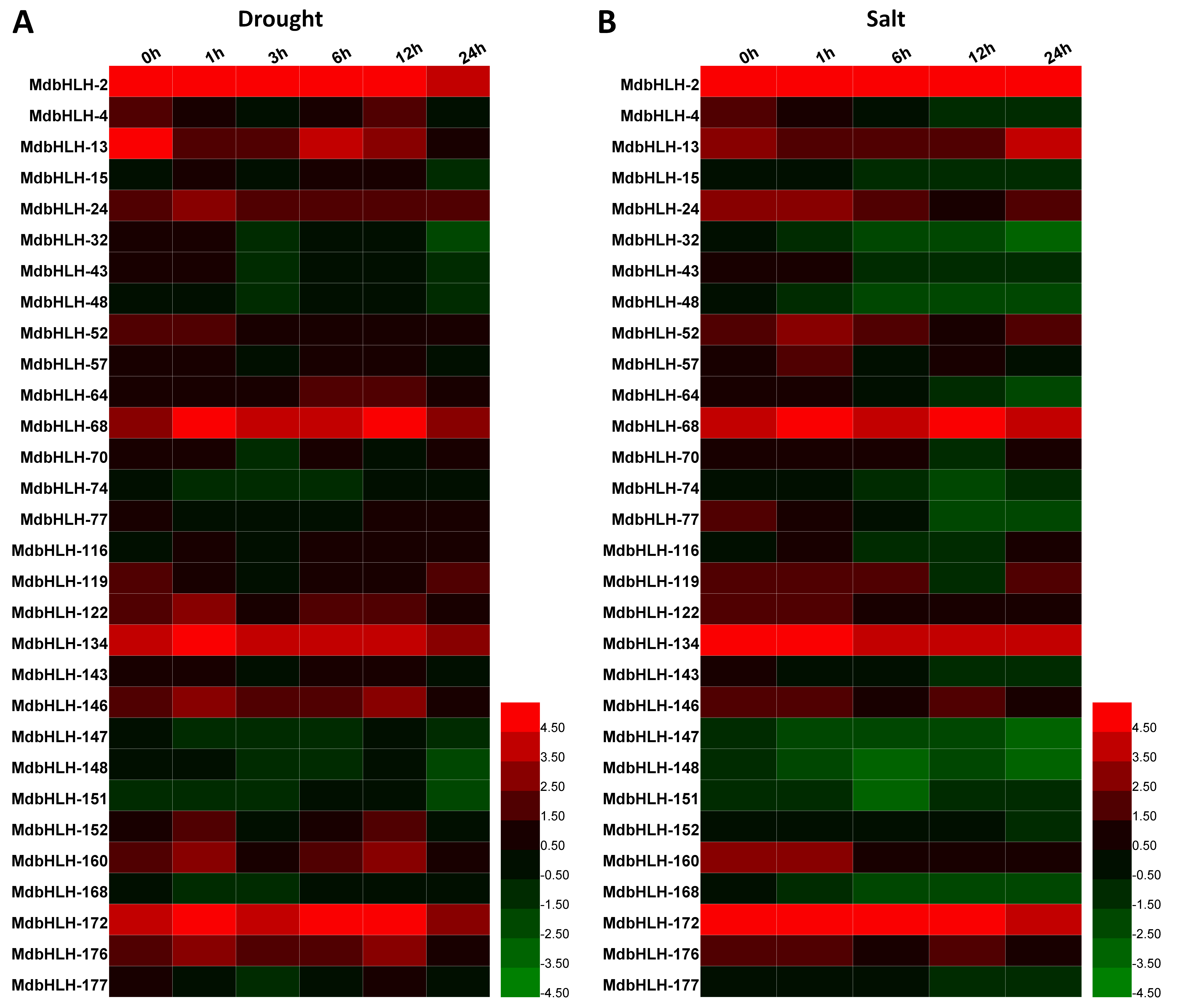

Supplement: Supplementary Figure S6 — Expression analysis of selected MdbHLH genes. Analysis (2−ΔCT method) of 30 MdbHLHs expressed in response to induced drought (PEG6000) (A) or salt stress (NaCl) (B). [file Image6.jpg]

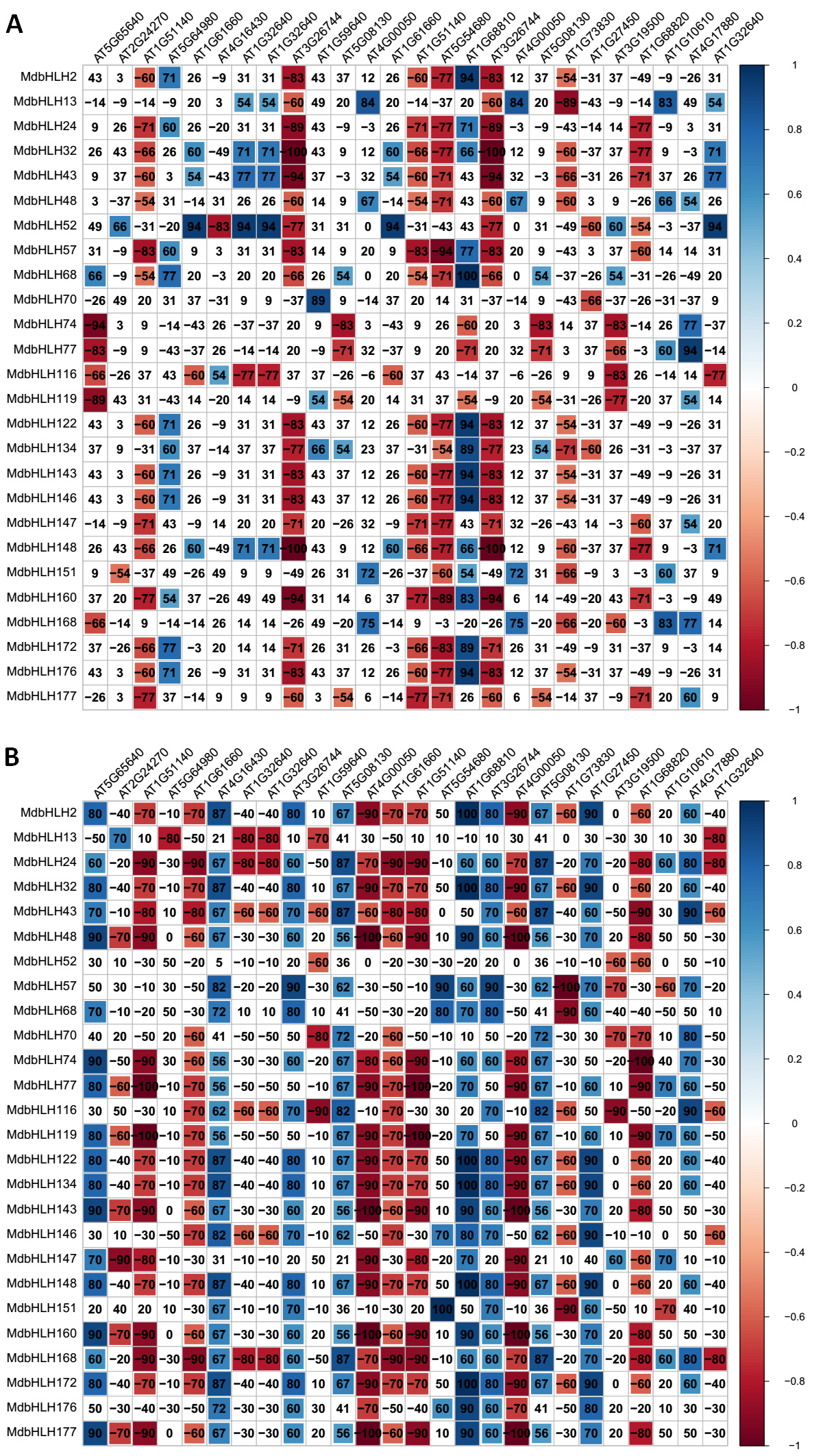

Supplement: Supplementary Figure S7 — Correlation analysis between the expression patterns of selected MdbHLH genes and MdbHLH orthologs in Arabidopsis, under drought (A) or salt (B) conditions. The value of spearman correlation coefficent in the heatmap was multiplied by one hundred. Positive or negative correlation relationships between the gene expression patterns are indicated with positive or negative values. Color depth (blue or red) of the grids corresponds to the value. [file Image7.JPEG]
